# Cannabis: harmless recreation or dangerous drug?

**DOI:** 10.1093/eurpub/ckz080

**Published:** 2019-05-23

**Authors:** Peter Allebeck

**Affiliations:** Department of Public Health Sciences, Karolinska Institutet, Sweden

‘It doesn’t work’. This is a consistent message in the series of Viewpoint contributions on cannabis policy published in this issue. They are based on a round table discussion held during the EPH conference in Ljubljana 2018, where we were updated on the situation regarding cannabis production, distribution and use in some European countries. Another message was that a balanced discussion is lacking in most countries. Strong ideological motives with political undertone is driving the debate, and public health considerations are missing.

So what are the public health aspects of cannabis? It is well documented that regular cannabis use, especially when more potent forms of the drugs are used, is detrimental to health. Reduced cognitive function, dependence, psychosis are the more well-known consequences, while increased risk of certain cancer forms, and risk of transition to ‘hard drugs’ are still debated.^1^^,^^2^ It is easy to say that alcohol causes more harm to health than cannabis, and even than drug use overall, including ‘hard drugs’. But another illegal substance in the EU, smokeless tobacco (‘snus’) is much less harmful than cannabis. So the regulation of dependency prone substances is seldom based solely on level of harm caused.

The Viewpoint papers, and other examples given at the roundtable discussion, illustrate that although cannabis is an illegal substance in all European countries, the character of its legal status varies strongly. And perhaps implementation and control vary even more. Although much is said about ‘legalization’, in no country is cannabis allowed to be marketed as bread and milk. Although in practice, a few places in the world are close to such a situation, all countries and communities have a variety on policies covering all the chain from cultivation to use, over production, distribution and marketing. For ‘consumers’, and perhaps those who want to start business, it is easy to find internet sites like a ‘travellers’ guide’, where the legal status in different countries are tabulated along these different dimensions.

It is clear that some of the policies applied in some countries are counterproductive. Users at the end of the chain are caught and get a criminal record. The illegal status drives a black market, is the argument,^3^ and it may be the case that police resources should be better used elsewhere. But it has not been shown that the ‘legalization’ started in some places have reduced criminality or black market. A policy brief from the Alice Rap project, that strongly advocates the argument that prohibition does more harm than good, even recognizes that the health effects of liberalization are unknown.

While the ‘war against drugs’, and a hard policy against drug users, has not shown to reduce the harmful effects of cannabis use, legalization in the meaning of opening up for commercial interest must be considered a dangerous way to go. In a recent paper, DiFiori and collaborators showed that sites in Europe with high availability of cannabis, and particularly in stronger forms, is related to increased risk of psychosis at population level, which has not been shown before.^4^ Increased availability, and especially combined with strong marketing, has so far always resulted in increased use, and increased level of harm (alcohol, tobacco, gambling, etc.).

What about medical use? For some reason, use of cannabinoids for treatment of illness has become a political issue, whereas it is normally up to medical product agencies to assess effectiveness and forms of licensing, dispensing, cost, etc. Opiates and amphetamines are well established pharmaceutical drugs for specific purposes, but medical use and indications for use are something very distinct from use of these substances as ‘street drugs’. Medical treatment policies are not normally debated and voted in parliamentary bodies, and it would be good to have the debate on recreational use clearly distinct from medical use.

While occasional recreational use cannot be considered harmful to health, the health and social consequences of cannabis use should not be underestimated. Regular use among young persons, and especially products of high potency, is a definite public health problem. A public health approach must use classical methods of prevention, including medical and social services, schools, community leaders, etc. There are good examples of local police collaborating with social services and community leaders. But prevention of substance use should not be mainly a task for police and criminal justice. And opening up new markets with economic incentives in production and distribution is hardly a sensible public health approach.
